# Editorial: New directions in forensic psychology: applying neuropsychology, biomarkers and technology in assessment & intervention

**DOI:** 10.3389/fpsyg.2024.1479498

**Published:** 2024-09-24

**Authors:** Joan E. van Horn, Märta Wallinius, Yvonne H. A. Bouman, Patrice Renaud, Josanne D. M. van Dongen

**Affiliations:** ^1^Research Department de Waag, Center for Outpatient Treatment, Utrecht, Netherlands; ^2^Evidence-Based Forensic Psychiatry, Department of Clinical Sciences Lund, Psychiatry, Lund University, Lund, Sweden; ^3^Center of Ethics, Law and Mental Health, Institute of Neuroscience and Physiology, The Sahlgrenska Academy, University of Gothenburg, Gothenburg, Sweden; ^4^Department of Research, Regional Forensic Psychiatric Clinic, Växjö, Sweden; ^5^Department of Research, Transfore, Institute for Forensic Mental Health Treatment, Deventer, Netherlands; ^6^Université du Québec en Outaouais and Institut National de Psychiatrie Légale Philippe-Pinel, Montréal, QC, Canada; ^7^Department of Psychology, Education and Child Studies, Erasmus University Rotterdam, Rotterdam, Netherlands

**Keywords:** forensic, virtual reality, wearables, neuropsychology, biomarkers

## General information

The forensic field has recently witnessed a growing interest in neuropsychology, wearables, and VR technology. These emerging areas promise to enhance the diagnosis and treatment of various forensic subpopulations. Our Research Topic, “*New directions in forensic psychology: applying neuropsychology, biomarkers and technology in assessment & intervention*,” encompasses 15 papers detailing the latest advancements in these domains. Through these contributions, we aim to encourage further research.

Most papers focus on the application, viability, and impact of virtual reality (VR) across various forensic groups. While submissions on wearables and neuropsychology were less frequent within this Research Topic, the included studies are of significant value. Eighty-nine contributing authors hail from diverse global regions, including Italy, Canada, and Colombia, with a notable concentration from the Netherlands and Sweden. We value the international diversity of the submissions, which mirrors the widespread global interest in cutting-edge diagnostic and therapeutic options in forensic mental health care.

The Research Topic is organized into four thematic sections: neuropsychology and neurofeedback, wearables, qualitative VR studies, and quantitative VR studies. The following sections provide a concise overview of the respective papers included.

## Neuropsychology and neurofeedback

Four papers cover forensic neuropsychology, neurofeedback, and a critical reflection of this use via the possible implementation of neurorights. The paper by Hutten et al. encompasses a Delphi consensus study on the neuropsychology of aggression. It elucidated the professionals' view on using neuropsychological tests to study different types of aggression and their diverse aspects, which are included in the RDoC framework. Balestrino et al. studied the usefulness of a handwriting test in assessing cognitive impairments. They found that a single score, the COGnitive Impairment Through handwriting (COGITAT) score, reliably assessed the writer's cognitive state. Because previous research has shown that psychological treatment, such as Cognitive Behavioral Therapy, is ineffective for all forensic clients, Hendriks et al. studied the feasibility and usability of neuromodulation training in a forensic outpatient clinic. They found that the training was rated sufficiently usable and feasible by patients and their therapists. Given the emergence of neurotechnological applications in forensic psychiatry and criminal law more broadly, such as the above-described neurofeedback treatment, there is a call for the implementation of so-called neurorights. Díaz Soto and Borbón evaluated the status of this matter and concluded that, although the interpretation of the current human rights should be made in such a way as to protect the dignity of the accused or client, new neurorights may offer reduced protection of human rights.

## Wearable technology

Two notable studies investigated the role of wearable technology in forensic psychiatry. de Looff et al. conducted a randomized crossover trial to evaluate the usability and acceptance of four wearable devices among forensic psychiatric patients and staff. Their findings revealed that while fitness trackers like Fitbit and Garmin were more user-friendly, none of the devices met international usability standards, highlighting the need for improved gamification and motivational features. In a complementary study, ter Harmsel et al. assessed the Sense-IT bio cueing app's effectiveness when added to Aggression Regulation Therapy (ART) for forensic outpatients. Although the app increased interoceptive awareness in most participants, its impact on aggression and emotion regulation was inconsistent.

These studies contribute valuable insights into the forensic field, emphasizing the potential of wearable technology to enhance therapeutic outcomes. Key lessons include the critical importance of usability, personalized interventions, and seamless integration into therapy for successful adoption. Both studies demonstrate solid methodologies, though limitations such as small sample sizes and the need for better algorithm validation highlight areas for future improvement. Overall, these studies underscore the necessity of tailoring technology-based interventions to individual needs for effective forensic psychiatric treatment.

## Qualitative studies on VR interventions

The implementation of Virtual Reality in forensic psychiatric treatment is relatively new. Several authors have applied qualitative methods to explore the experience of patients and clinicians with VR applications such as DEEP, Virtual Reality Aggression Prevention Training (VRAPT), or Triggers and Helpers. Two out of three studies had been conducted in the Netherlands (Klein Haneveld et al.; Kouijzer et al.) and one in Sweden (González Moraga et al.).

Whereas VRAPT and Triggers and Helpers are blended applications in which roleplaying assists patients in improving awareness and social skills, e.g., reduction of aggressive behavior, DEEP is an application in which a patient practices deep breathing in a gamified biofeedback underwater world.

These three studies used interviews to explore the participants' experiences and seek answers to the questions of for whom and when these applications are helpful in forensic psychiatric treatment. In the DEEP study, the authors sought answers to which application method would suit whom best. Apart from suggestions on improving immersiveness, ideas on implementing DEEP in clinical practice emerged. The study by Kouijzer et al. focused on implementation and used Triggers and Helpers as a showcase. Patient characteristics must be considered when deciding to whom this method should be offered, and continuously assisting clinicians when they use VR seems a vital necessity. The Swedish qualitative evaluation of patients' experience with VRAPT also highlighted the need to thoroughly implement innovative treatments such as VR and personalize treatment goals for which VR can be used.

## Quantitative studies on VR interventions

There were six quantitative studies, most from Europe (the Netherlands, Sweden) and one from Canada. All studies except one tested different VR interventions in clinical forensic settings, while one investigated a chatbot developed for risk assessment training. Given the state of the field, the quantitative studies overall had a feasibility and effect approach and described interventions with the need for continued development and evaluation. Common findings were that all users' attitudes toward technology-driven interventions were generally positive and that outcomes depended on successful implementation in interventional settings.

Several studies focused on the treatment of aggression regulation for either forensic psychiatric patients or imprisoned offenders, evaluating either a method specifically designed for VR-assisted treatment only (Virtual Reality Aggression Prevention Training) or comparing outcomes from a treatment (Responsive Aggression Regulation Therapy) being delivered either in virtual environments or in real life settings. The results of the two studies presenting longitudinally followed outcomes over time were promising, with decreased levels of anger, aggression, and emotion regulation maintained over follow-up. Two studies focused on assessment, both with an experimental design, where one investigated the feasibility of paranoia assessment in virtual environments, and the other determined acceptance and trust of students on chatbot-assisted risk assessment training. For both studies, not only the technology but also the user characteristics seemed necessary for the usefulness of the assessment. So far, we cannot replace standard training and assessment with technology-driven versions. Finally, a study used a VR-based intervention to prepare forensic psychiatric patients for authorized leave, and the potential of VR to increase patient motivation and reduce stress was evident.

In summary, many of the quantitative studies on this Research Topic benefited from the characteristics of VR, which facilitates exposure to specific environments in preparation for real-life occurrences. However, it is evident that much is left to investigate and that further developments, especially concerning individual tailoring, are needed. The current findings can guide clinicians and researchers in forensic settings in their coming ventures on these matters.

## General conclusion

The collective insights from the 15 papers featured in our Research Topic highlight the transformative potential of integrating neuropsychology, biomarkers, and advanced technology into forensic psychology. Through diverse methodologies, these studies illustrate how neuropsychological assessments, wearable devices, and virtual reality (VR) interventions can enhance diagnosis, treatment, and overall therapeutic outcomes in forensic populations.

Key lessons from these studies include the importance of feasibility and usability in implementing new technologies. For instance, neuromodulation and neurofeedback have shown initial promise in addressing impulse control issues, but practical challenges must be addressed to ensure broader application. Similarly, using wearables, while beneficial in some cases, reveals that user engagement and device adaptability are crucial for sustained success. The adoption of VR in forensic settings stands out for its ability to simulate realistic scenarios, providing a safe environment for behavior assessment and skills training. Studies on VR-assisted aggression treatment, for example, indicate positive patient experiences and potential for improving therapeutic outcomes. However, these interventions require a careful introduction to avoid exacerbating psychological distress, especially in vulnerable populations.

Integrating these technologies necessitates a multifaceted approach, combining rigorous scientific validation with practical considerations. Future research should focus on refining these tools, tailoring interventions to individual needs, and ensuring ethical standards are met, particularly concerning mental privacy and dignity. The findings presented serve as a valuable guide for future research and clinical practices, emphasizing the need for tailored, technology-driven interventions to serve forensic populations better.

